# Hemodialysis-induced renal perfusion decline: unraveling the pathophysiological mechanisms linking intradialytic circulatory stress to residual renal function loss

**DOI:** 10.3389/fphar.2025.1648608

**Published:** 2025-10-20

**Authors:** Dayang Xie, Jiaming Tan, Qingtao Zhang, Qian Yu, Yiqin Wang, Yujin Wang, Li Gao, Liyuan Yan, Jianhui Zhou, Nan Li, Guangyan Cai

**Affiliations:** ^1^ Senior Department of Nephrology, Chinese PLA General Hospital, State Key Laboratory of Kidney Diseases, National Clinical Research Center for Kidney Diseases, Beijing Key Laboratory of Medical Devices and Integrated Traditional Chinese and Western Drug Development for Severe Kidney Diseases, Beijing Key Laboratory of Digital Intelligent TCM for the Preventionand Treatment of Pan-vascular Diseases, Key Disciplines of National Administration of Traditional Chinese Medicine(zyyzdxk-2023310), Innovation Team and Talents Cultivation Program of National Administration of Traditional Chinese Medicine. (No: ZYYCXTD-D-202402), Beijing, China; ^2^ Department of Ultrasound, First Medical Center of Chinese PLA General Hospital, Beijing, China

**Keywords:** hemodialysis, residual renal function, renal perfusion, perfusion index, ultrafiltration

## Abstract

**Background:**

Residual renal function (RRF) plays a critical role in quality of life and survival in hemodialysis (HD) patients but characteristically declines after the initiation of HD. Owing to incomplete understanding of the pathophysiology underlying RRF decline, protective strategies remain limited. The aim of this study was to explore the dynamic changes of renal perfusion in incident HD patients with preserved RRF during dialysis sessions and to provide new strategies for RRF preservation.

**Methods:**

This prospective cohort study enrolled 30 incident HD patients with preserved RRF. Renal perfusion was serially assessed using contrast-enhanced ultrasonography (CEUS) at three time points during the HD session: pre-dialysis baseline, intradialytic phase (3 h post-initiation), and post-dialysis recovery phase (15 min after session completion). Renal perfusion was quantified using the CEUS-assessed perfusion index (PI). The primary outcome measure was the PI.

**Results:**

During hemodialysis sessions, the PI as a surrogate marker of renal perfusion decreased by 17.53% (P < 0.001), which exhibited a negative correlation with ultrafiltration (UF) rates (Spearman’s r = −0.770, P < 0.001), but not with other variables such as sex, age, body mass index (BMI), blood pressure (BP), estimated glomerular filtration rate (eGFR), hemoglobin, or albumin levels.

**Conclusion:**

This study demonstrates that incident HD patients experience an acute decrease in renal perfusion during hemodialysis, which is negatively correlated with mean UF rates. This finding may represent a crucial step toward elucidating the pathophysiology of hemodialysis-mediated RRF decline.

**Clinical trial registration:**

clinicaltrials.gov, identifier (NCT07003828).

## 1 Introduction

The persistence of RRF is crucial for dialysis patients (Tanriover et al., 2022), as it not only facilitates higher clearance of solutes, maintenance of fluid balance, and control of electrolytes but also exerts beneficial effects on inflammation (de Sequera et al., 2017; Raikou et al., 2018), anemia (Wang and Lai, 2006; Wang et al., 2002), malnutrition (Lin et al., 2018; Zhou et al., 2018), cardiac function (Raikou et al., 2018; Wang and Lai, 2006), diabetes mellitus (DM) (Perl and Bargman, 2009), obesity (Obi et al., 2018), and changes in gut microbiota (Cupisti et al., 2021). More importantly, it is strongly correlated with reduced mortality and improved quality of life (Perl and Bargman, 2009; Li et al., 2019; Okazaki et al., 2023). Although only minimal RRF is retained in patients initiated on maintenance dialysis, this is sufficient to make a significant contribution to the removal of potential uremic toxins since renal filtration is continuous, as opposed to the 12 h per week that the patient is undergoing hemodialysis (Adequacy of dialysis and nutrition, 1996; Shemin et al., 2001; Bonomini et al., 1976; Merkus et al., 2000; Toth-Manikowski et al., 2020). In addition, RRF allows for the clearance of larger molecules, such as β2-microglobulin, which dialysis filters cannot remove. In reality, most incident HD patients still retain significant RRF at dialysis initiation (Kjaergaard et al., 2011; van der Wal et al., 2011). According to the United States Renal Data System, only 15% of individuals starting dialysis in 2020 had an eGFR <5 mL/min/1.73 m2 (United States Renal Data System, 2022). Unfortunately, RRF typically declines after HD initiation, with even 25%–67% of incident HD patients developing anuria by 10 months (Moist et al., 2000; Jansen et al., 2002; McKane et al., 2002; Lin et al., 2009; Fernández-Lucas et al., 2012). The rate of RRF decline was significantly faster in hemodialysis patients compared to peritoneal dialysis (Moist et al., 2000; Jansen et al., 2002), and more frequent HD sessions correlated with accelerated RRF decline (Obi et al., 2016; Daugirdas et al., 2013). Owing to poor understanding of the pathophysiology underlying RRF decline, protective strategies remain limited.

Previous research investigating the pathophysiology underlying RRF decline in HD patients has confirmed that decreased renal perfusion (DRP) represents the first key step toward understanding the mechanisms of RRF loss in this population (Marants et al., 2019). However, due to concerns regarding the potential effects of contrast-induced nephropathy, the study restricted its cohort to patients with already low RRF. Whether similar intradialytic renal perfusion changes also occur in patients with a preserved RRF remains unclear. In fact, studies on these patients are more clinically significant, as they retain potential for intervention, particularly during the early stages of dialysis. To address this knowledge gap, the aim of this study was to explore the changes in renal perfusion in incident HD patients during dialysis, to provide new strategies for RRF preservation.

CEUS is an effective method for real-time, noninvasive, accurate, and quantitative assessment of renal perfusion (Kalantarinia et al., 2009; Kogan et al., 2011). Currently, it has shown promising utility in the investigation of various kidney diseases (Wang and Mohan, 2016), including chronic kidney disease (CKD) (Dong et al., 2014), Acute kidney injury (AKI) (Kalantarinia, 2009; Schneider et al., 2013), and kidney transplantation (Wang et al., 2015; Jin et al., 2015). Compared with contrast-enhanced computed tomography (CECT) and contrast-enhanced magnetic resonance (CEMR), CEUS offers advantages including lower cost, shorter performance time, absence of ionizing radiation and nephrotoxicity, and repeatability even at the bedside (Granata et al., 2021; Atri et al., 2022), thereby making it ideal for serial renal perfusion monitoring in HD patients. As a CEUS intensity parameter, PI demonstrates high sensitivity in detecting reductions in renal perfusion, accurately reflects alterations in renal cortical microcirculation, and shows a positive correlation with eGFR (Schneider et al., 2012; Xu et al., 2024). Therefore, similar to previous studies quantifying renal microperfusion, we used the PI as a surrogate measure of renal perfusion (Garessus et al., 2022; Damianaki et al., 2024).

## 2 Materials and methods

### 2.1 Participants

A total of thirty patients from the First Medical Center of Chinese PLA General Hospital were consecutively enrolled in the study (as detailed in the Study Design section) after providing written informed consent. Inclusion criteria were: (1) patients aged ≥18 years old with end-stage renal disease (ESRD); (2) incident HD patients, defined as individuals with a hemodialysis duration of ≤3 months since initiation; (3) preserved RRF defined as urinary output >500 mL/24 h or eGFR >3 mL/min/1.73 m^2^; (4) use of central venous catheter for dialysis access. Exclusion criteria: (1) known allergy to Sonovue^®^; (2) vascular access dysfunction; (3) combined peritoneal dialysis; (4) renal vascular disease; (5) severe cardiopulmonary disease; (6) active infection or malignancy; (7) communicable diseases; (8) pregnancy or breastfeeding; (9) participation in other clinical trials. The study was approved by the Ethics Committee of Chinese PLA General Hospital (Approval No. S2024-294-01) and was conducted in accordance with the approved protocols, Good Clinical Practice guidelines, and relevant regulatory requirements.

### 2.2 Study design

This prospective observational study enrolled 30 incident HD patients meeting predefined inclusion criteria. CEUS examinations were performed at three predetermined time points during each HD session: immediately before, 3 h after HD initiation, and 15 min post-dialysis. The primary outcome was the PI measured by CEUS. We prospectively collected clinical and laboratory parameters for all participants, including sex, age, body mass index (BMI), comorbidities, 24-h urine volume, serum creatinine, blood urea nitrogen, hemoglobin, serum albumin, and eGFR. The dialysis-related variables were recorded, including UF and dialysis-related adverse events. Intradialytic blood pressure was measured at 0 (pre-dialysis), 60, 120, 180, and 240 min (post-dialysis). To minimize confounding factors, standardized HD protocols were implemented using FX60 hemodialyzers (FX Class Capillary Dialyzers; Fresenius Medical Care) with fixed operational parameters: 4-h session duration, dialysate temperature maintained at 36.5 °C, and blood flow rate set to 220 mL/min. Because Sonovue^®^ has a short effective imaging time, which is rapidly eliminated via pulmonary circulation, with >80% pulmonary excretion within 2 min and near-complete clearance by 15 min post-injection (Junhong and Wen, 2024), we evaluated only one kidney (left kidney).

### 2.3 CEUS image acquisition and analysis

We performed all measurements using the Mindray Resona I9 device with a dedicated abdominal probe (3–5 MHz), and used Sonovue^®^ (Bracco SpA, Milan, Italy) as the contrast agent (SonoVue, a pure blood pool contrast agent, consists of microbubbles with an average diameter of 2.5 μm, mirroring the size of red blood cells. It is primarily cleared via the lungs and liver and is not removed by HD.) (Junhong and Wen, 2024). All CEUS examinations were performed according to the published protocols by an experienced sonographer (M.P.) with >10 years CEUS experience (Junhong and Wen, 2024). The left kidney was selected for renal perfusion measurements in all participants. With the patient positioned in the right lateral decubitus position, ultrasound scanning of the left kidney was performed to acquire the maximal coronal section through the renal hilum. Sonovue^®^ was prepared and used in strict accordance with manufacturer guidelines: 5 mL of 0.9% sterile sodium chloride solution was introduced into the vial prior to use, followed by vigorous shaking for 20 s to achieve homogeneous microbubble dispersion. During the procedure, 1.2 mL of contrast agent solution was administered as a bolus injection via the left antecubital vein, followed by a 5 mL saline flush. Timed acquisition of a 3-min renal CEUS video commenced immediately upon contrast agent injection, with patients instructed to maintain quiet breathing throughout the imaging sequence. Following administration, patients were kept under close medical observation for at least 30 min to ensure safety and to monitor for any adverse reactions. Image analysis was performed using integrated into the Mindray ultrasound system. The region of interest (ROI) was positioned within the mid-renal cortical area, with a standardized size of 20–30 mm^2^. Triplicate measurements were averaged to generate time-intensity curves (TICs), with contrast enhancement parameters including PI methodically recorded. To ensure methodological consistency, both spatial placement and dimensional parameters of ROIs were rigorously standardized across all study participants.

### 2.4 Statistical analyses

This is an exploratory study and there are inadequate data exist to perform a meaningful sample size calculation. We pragmatically selected an enrollment target of 30 participants. This sample size is comparable with statistician’s recommendations (Whitehead et al., 2016).

Statistical analysis was performed using SPSS Statistics version 27.0. Continuous variables were represented by mean ± standard deviation, categorical variables were expressed as a percentage. Data were analyzed using repeated measures ANOVA with post hoc t tests (with Bonferroni correction). Associations between variables were assessed using the Pearson product-moment correlation coefficient, if variables deviated from normality, the Spearman correlation coefficient was utilized. P values <0.05 were considered statistically significant.

## 3 Results

### 3.1 Clinical characteristics of study population

A total of 30 incident HD patients were included. The study subjects’ demographic, clinical, and biochemical characteristics are presented in Table 1. The mean age was 53.43 ± 15.10 years, with 80% male participants. The mean dialysis vintage was 15.83 ± 4.33 days, with a range from 9 to 26 days. The prevalence of comorbidities included 56.67% DM, 93.33% hypertension, 23.33% cardiovascular disease, 53.33% peripheral vascular disease. The eGFR was 6.16 ± 2.36 mL/min/1.73 m^2^, mean urinary output was 1,305 ± 539.53 mL/24 h, and mean UF rate was 0.10 ± 0.05 mL/min/kg.

**TABLE 1:** Baseline characteristics of study population.

Characteristics	Values
Age (years)	53.43 ± 15.10
Men [n (%)]	24 (80)
Weight (kg)	72.03 ± 12.05
BMI (kg/m^2^)	25.61 ± 3.91
eGFR (mL/min/1.73 m^2^)	6.16 ± 2.36
Urinary output (mL/24 h)	1,305 ± 539.53
Dialysis vintage (days)	15.83 ± 4.33
Hemoglobin (g/L)	92.97 ± 16.42
Aldosterone (g/L)	35.28 ± 5.46
Intradialytic weight gain (mean, kg)	1.87 ± 1.03
Mean ultrafiltration (UF) rate (mL/min/kg)	0.10 ± 0.05
Primary kidney disease [n (%)]	
Chronic glomerulonephritis	16 (53.33)
Diabetic kidney disease	10 (33.33)
Hypertensive nephrosis	2 (6.67)
Other (or unknown)	2 (6.67)
Comorbidity [n (%)]	
Coronary artery disease	7 (23.33)
Peripheral vascular disease	16 (53.33)
Diabetes	17 (56.67)
Hypertension	28 (93.33)

Continuous variables are presented as mean ± SD, while non-continuous variables are presented as number (percentage). The ultrafiltration (UF) rate [ml/min/kg], calculated as total UF, volume [ml] divided by the length of hemodialysis (HD) session [min] and further divided by the patient’s body weight [kg]. The 24-h urine volume range was 650–2,850 mL/24 h. BMI, body mass index.

### 3.2 Renal perfusion

**FIGURE 1:**
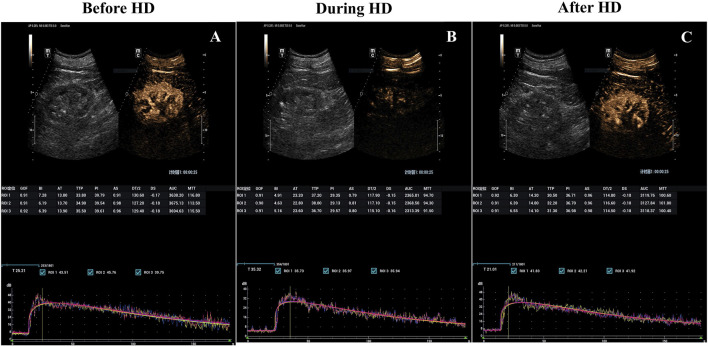
Hemodialysis-induced decrease in kidney blood flow visualized with parametric renal perfusion maps. Renal blood flow at baseline **(A)**, 3 h into **(B)** and 15 min after hemodialysis sessions **(C)** for the incident hemodialysis patients with a preserved RRF. RRF, Residual renal function.

**FIGURE 2:**
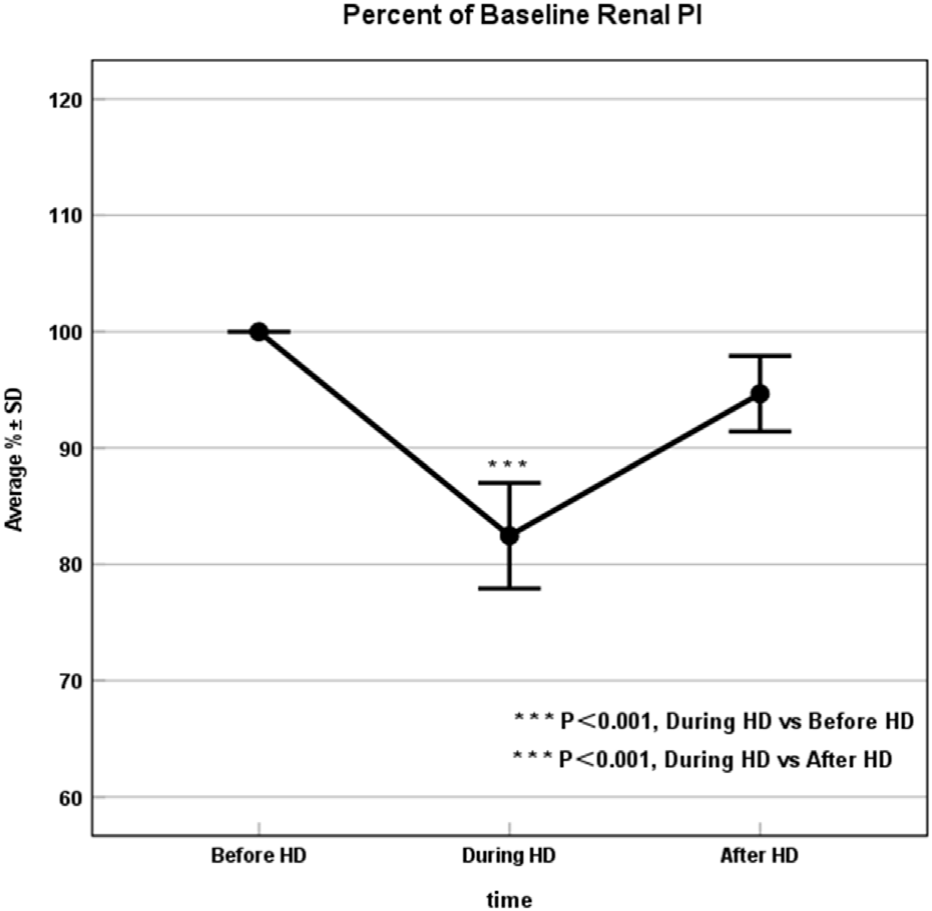
Renal Perfusion significantly declined during HD. Percent of baseline the renal perfusion before, 3 h into, and after dialysis, where results are given as average ± SD. The drop in renal perfusion during HD was statistically significant compared with pre- and post-HD blood flow values (P < 0.001). HD, Hemodialysis.

All enrolled patients (30/30, 100%) successfully underwent CEUS, without any microbubble-related adverse events observed. Average baseline PI was 38.37 ± 5.23 (mean ± SD). At peak stress (3 h after HD initiation), average PI dropped to 82.47% ± 12.43% of baseline. After HD, average PI recovered to 94.67% ± 8.74% of baseline (Table 2; Figures 1, 2). Repeated measures ANOVA with post hoc testing demonstrated that the intradialytic PI drop was statistically significant compared with pre- and post-HD (P < 0.001).

**TABLE 2:** The changes in Perfusion Index (PI) and Blood Pressure (BP) during Hemodialysis.

Parameter	Before HD	During HD	After HD	P (before HD vs. during HD)
PI	38.37 ± 5.23	31.58 ± 6.29**	36.34 ± 6.18	<0.001
SBP (mmHg)	147.33 ± 15.89	135.37 ± 17.02*	148.2 ± 20.63	0.012
DBP (mmHg)	77.40 ± 11.11	73.30 ± 11.55	78.53 ± 11.35	0.079
MAP (mmHg)	100.71 ± 10.81	93.99 ± 12.17*	101.76 ± 13.00	0.010

Data are presented as mean ± SD., The PI, and BP, were measured at three times during each HD, session: immediately before, 3 h into, and 15 min after dialysis. *P < 0.05 vs. Baseline; **P < 0.01 vs. Baseline. PI, perfusion index; BP, blood pressure; SBP, systolic blood pressure; DBP, diastolic blood pressure; MAP, mean arterial pressure.

### 3.3 Relationship to dialysis stress factors

During dialysis sessions, none of the 30 patients experienced intradialytic hypo-tension (IDH, defined as a symptomatic drop in systolic blood pressure [SBP] >20 mmHg) or other dialysis-related adverse events. SBP and mean arterial pressure (MAP) dropped significantly during HD to 92.59% ± 12.61% (P = 0.012)and 93.99% ± 12.17% (P = 0.01)of baseline, respectively, before both recovering to 100.87% ± 11.44% and 101.76% ± 13.00% of baseline post-HD. Although diastolic blood pressure (DBP) showed a similar trend, the intradialytic change to 95.75% ± 14.88% of baseline was not significant (P = 0.079). However, no significant correlations were observed between alterations in SBP, DBP, or MAP and changes in PI (P > 0.05). Similarly, neither blood pressure variability parameters—including SBP standard deviation (SBP-SD), SBP co-efficient of variation (SBP-CV), DBP standard deviation (DBP-SD), nor DBP coefficient of variation (DBP-CV)—demonstrated any association with renal perfusion modifications (P > 0.05) (Table 3).

**TABLE 3:** Univariate Correlations (Spearman) Between Clinical Parameters and the Change in PI (ΔPI, During HD vs. Before HD).

Parameter	R	P-value
Sex	0.31	0.084
Age	−0.288	0.123
Weight	0.165	0.384
BMI	0.059	0.759
eGFR	0.022	0.907
Hemoglobin	0.248	0.187
Albumin	0.325	0.08
Mean ultrafiltration (UF) rates	−0.770	<0.001
SBP		
ΔSBP/pre-SBP	0.313	0.093
SD	0.235	0.212
CV	0.183	0.333
DBP		
ΔDBP/pre-DBP	0.072	0.706
SD	−0.237	0.208
CV	−0.287	0.124
ΔMAP/pre-MAP	0.202	0.283

Blood pressure change (ΔBP), which refers to the pre-dialysis blood pressure (0 min) minus the intradialysis (180 min) blood pressure. The percent change in blood pressure (ΔBP/pre-BP), calculated as ΔBP, divided by pre-dialysis blood pressure and multiplied by 100%. SD, represents the standard deviation of blood pressure measurements at 0, 60, 120, and 180 min. CV, was the SD, divided by the mean and multiplied by 100%; Mean arterial pressure (MAP) was calculated using the standard formula: MAP = (SBP + 2 × DBP)/3. BP, blood pressure; SBP, systolic blood pressure; pre-SBP, pre-dialysis systolic blood pressure; DBP, diastolic blood pressure; pre-DBP, pre-dialysis diastolic blood pressure.

**FIGURE 3:**
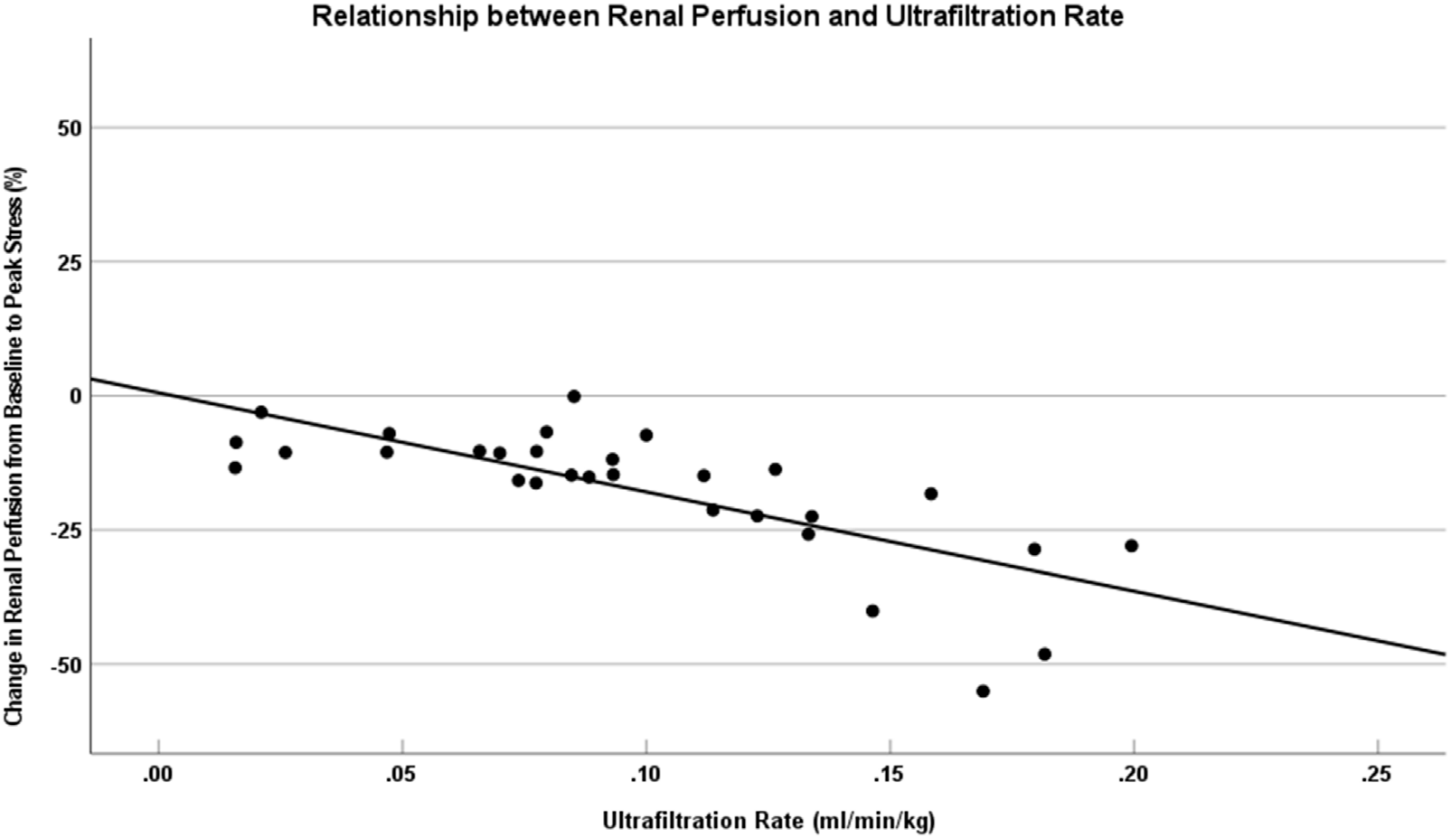
DRP was associated with higher UF rates during HD. This figure shows that change in the kidney perfusion from baseline to peak stress versus UF rate for HD patients. The solid lines represent data trendlines for the renal perfusion. The UF rate was associated with a larger drop in renal perfusion from baseline to peak stress (r = −0.770, P < 0.001). DRP, Decreased renal perfusion; UF, Ultrafiltration; HD, Hemodialysis.

In the present study, the mean UF rate was 0.10 ± 0.05 mL/min/kg, showing a significant inverse correlation with DRP (Spearman’s r = −0.770, P < 0.001) (Figure 3). This negative correlation between UF rate and DRP remained robust upon validation with 1,000 bootstrap resamples (95% CI: 0.905 to −0.460). No significant associations were observed between PI drop and other variables, including sex, age, BMI, eGFR, hemoglobin, or albumin levels. (P > 0.05; Table 3).

## 4 Discussion

This study demonstrated that incident HD patients with a preserved RRF also experience an acute decrease in renal perfusion during dialysis, which was negatively associated with the mean UF rate but not with other variables such as sex, age, BMI, blood pressure, eGFR, hemoglobin, or albumin levels. These important findings may provide a pathophysiologic explanation and potential preventative strategies for the characteristically rapid decline of RRF in HD patients.

As stated previously, RRF is critical for dialysis patients. RRF preservation not only increases hemodialysis adequacy and enhances better management of hemodialysis-related complications but also improves survival and the quality of life (Perl and Bargman, 2009; Li et al., 2019; Okazaki et al., 2023). Unfortunately, RRF characteristically declines after HD initiation, with even 25%–67% of incident hemodialysis patients developing anuria by 10 months (Moist et al., 2000; Jansen et al., 2002; McKane et al., 2002; Lin et al., 2009; Fernández-Lucas et al., 2012). Due to the incomplete comprehension of the pathophysiology underlying RRF decline, protective strategies are limited. Previous studies only focused on patients with already low RRF (Marants et al., 2019). Consequently, current understanding of the pathophysiological mechanisms underlying hemodialysis-induced RRF decline is largely limited to this population. By contrast, the pathophysiology underlying RRF decline of patients with preserved RRF—particularly those in the incident hemodialysis phase—remains substantially unexplored. In reality, studies of the latter hold greater clinical significance, as most incident HD patients retain significant RRF at dialysis initiation and have more important intervention value. This study is the first to explore the pathophysiological mechanisms underlying RRF decline in incident HD patients with preserved RRF. The results showed that hemodialysis induced a sharp 17.53% decline in renal perfusion among patients, which was consistent with findings from prior studies on patients with already low RRF (Marants et al., 2019). This indicates that incident HD patients with preserved RRF similarly experience a sharp decline in renal perfusion during dialysis, thereby greatly increasing the risk of renal ischemic injury. As patients with ESRD undergo HD three to four times weekly, the repetitive renal hypoperfusion occurring in each session may increase the risk of progressive accumulation of kidney tissue damage. This may provide a plausible explanation for the gradual decline in urine output after dialysis initiation. This study focused on incident HD patients with a preserved RRF. To minimize iatrogenic injury to RRF, CEUS was employed for renal perfusion assessment. Although the current study provides less detailed mechanistic insight into perfusion dynamics than the CECT-based study by Marants et al. (which employed absolute perfusion measures, a controlled crossover design, and dialysate cooling interventions), CEUS offers significant advantages in terms of safety and clinical feasibility. These characteristics position CEUS as a modality with strong translational potential for routine implementation in dialysis units.

Previous studies on patients with already low RRF found that decreased renal perfusion was associated with higher mean ultrafiltration rates during HD (Marants et al., 2019). Similar conclusions have been drawn in this study, and also confirming previous observations that higher ultrafiltration rates (UFR) were associated with more rapid loss of RRF among patients receiving regular HD (Lee et al., 2020). These findings suggest that UF-induced ischemic injury may be a key factor in progressive RRF loss in HD patients. In clinical practice, optimizing UF rates could serve as a therapeutic strategy to ameliorate HD-induced renal tissue damage.

In the present study, none of the patients experienced IDH during dialysis. Even so, a significant decrease in renal perfusion was observed, consistent with the findings of Marants et al. (Marants et al., 2019). Imaging studies of the heart (Burton et al., 2009) and brain (MacEwen et al., 2018) during dialysis further support this phenomenon, suggesting that clinically significant end-organ ischemia may occur during hemodialysis even in the absence of overt hypotension. These results imply that reduced renal perfusion may arise independently of IDH. This study also discovered that SBP and MAP decreased significantly during HD. However, correlation analysis showed no association between blood pressure fluctuations and renal perfusion decline, which is consistent with observations from Marants et al. (Marants et al., 2019). Given that blood pressure was monitored episodically rather than continuously, the relationship between blood pressure changes and renal perfusion decline still needs to be verified in future studies. Additionally, no correlation was found between renal perfusion decline and eGFR. This observation may account for the similar degrees of renal perfusion reduction observed in patients with both low and preserved RRF. However, RRF represents a complex outcome influenced by multiple factors, including primary kidney disease type (e.g., diabetic vs. glomerular nephropathy), comorbidities (particularly diabetes and cardiovascular disease), medication regimens (e.g., RAS blockers, diuretics, NSAIDs), and systemic inflammation. Future studies should incorporate these variables through multivariate modeling or stratified subgroup analyses to enhance predictive validity.

## 5 Conclusion

In conclusion, this study illustrates that incident HD patients with preserved RRF experience an acute decrease in renal perfusion during dialysis, even in the absence of IDH. This indicates that these patients similarly experience ischemic insult, which is repeated during recurring dialysis sessions and may result in cumulative damage to kidney tissue. That is one of the reasons for the progressive RRF loss after dialysis initiation. However, larger-scale studies would be needed to validate these findings.

This study is the first to reveal the pathophysiological mechanisms of RRF loss in incident HD patients, offering insights that may guide preventive interventions to protect RRF. However, this research has several limitations. First, like all other clinical techniques used to quantify renal micro-perfusion, CEUS lacks a gold standard to validate our protocol, and consistent with previous studies, we examined only one kidney because Sonovue^®^ has a short effective imaging time, this may not fully represent bilateral renal perfusion or account for anatomical variability. Future protocols should either assess both kidneys or randomize kidney selection to improve generalizability. Second, during CEUS examinations, the bolus injection technique was employed in accordance with the prevailing domestic expert consensus. Although rigorous measures were implemented to control key confounders (including protocol standardization and respiratory coaching), residual confounding effects may persist due to the inherent limitations of the bolus injection technique and the complexity of respiratory motion. Therefore, to address these limitations, future research should focus on and explore the application value of various perfusion assessment methods, particularly the infusion technique with flash/reperfusion. Head-to-head comparisons across diverse clinical scenarios may help establish evidence-based protocols for standardized perfusion quantification. Third, in this study, the PI was selected as the primary metric based on domestic expert consensus and robust supporting evidence from high-quality studies. Given the inherent limitations of relying on a single parameter, future research will explore the combined application of multiple perfusion parameters, including mean transit time (MTT), to enable more comprehensive and precise assessment of renal perfusion. Lastly, this study is a cross-sectional study limited to a single HD session. Although the observed perfusion reduction demonstrated statistical significance, the absence of longitudinal follow-up or functional outcome measures (e.g., serial urine output, residual GFR) precludes definitive conclusions regarding long-term clinical relevance. Future studies will incorporate longitudinal CEUS measurements and correlate perfusion changes with RRF decline over time to establish true prognostic value. As an exploratory study with limited sample size, these findings require validation in future adequately powered trials and through multivariate modeling.

## Data Availability

The original contributions presented in the study are included in the article/supplementary material, further inquiries can be directed to the corresponding authors.
